# Inoculation Pneumonia Caused by Coagulase Negative *Staphylococcus*

**DOI:** 10.3389/fmicb.2019.02198

**Published:** 2019-10-04

**Authors:** Meng-meng Shi, Antoine Monsel, Jean-Jacques Rouby, Yan-ping Xu, Ying-gang Zhu, Jie-ming Qu

**Affiliations:** ^1^Department of Pulmonary and Critical Care Medicine, Ruijin Hospital, Shanghai Jiao Tong University School of Medicine, Shanghai, China; ^2^Institute of Respiratory Diseases, Shanghai Jiao Tong University School of Medicine, Shanghai, China; ^3^Multidisciplinary Intensive Care Unit, Department of Anesthesiology and Critical Care, La Pitié-Salpêtrière Hospital, Assistance Publique-Hôpitaux de Paris, Sorbonne University, Paris, France; ^4^Sorbonne Université, INSERM, UMR-S 959, Immunology-Immunopathology-Immunotherapy (I3), Paris, France; ^5^Biotherapy (CIC-BTi) and Inflammation-Immunopathology-Biotherapy Department (DHU i2B), Hôpital Pitié-Salpêtrière, AP-HP, Paris, France; ^6^Department of Pulmonary and Critical Care Medicine, Huadong Hospital, Fudan University, Shanghai, China

**Keywords:** inoculation pneumonia, coagulase-negative staphylococci, *Staphylococcus haemolyticus*, tumor necrosis factor-α, macrophage inflammatory protein-2

## Abstract

**Rationale:**

Although frequently retrieved in tracheal secretions of critically ill patients on mechanical ventilation, the existence of pneumonia caused by coagulase-negative staphylococci (CoNS) remains controversial.

**Objective:**

To assess whether *Staphylococcus haemolyticus* (*S. haemolyticus*) inoculated in mice’s trachea can infect normal lung parenchyma, increasing concentrations of *S. haemolyticus* were intratracheally administered in 221 immunocompetent mice.

**Methods:**

Each animal received intratracheally phosphate-buffered saline (PBS) (*n* = 43) or live (*n* = 141) or inactivated (*n* = 37) *S. haemolyticus* at increasing load: 1.0 × 10^6^, 1.0 × 10^7^, and 1.0 × 10^8^ colony forming units (CFU). Forty-three animals were sacrificed at 12 h and 178 were sacrificed at 36 h; 64 served for post-mortem lung histology, 157 served for pre-mortem bronchoalveolar lavage (BAL) analysis, and 42 served for post-mortem quantitative bacteriology of lung tissue. The distribution of biofilm-associated genes was investigated in the *S. haemolyticus* strain used in our *in vivo* experiment as well as among 19 other clinical *S. haemolyticus* strains collected from hospitals or nursing houses.

**Measurements and Main Results:**

Intratracheal inoculation of 1.0 × 10^8^ CFU live *S. haemolyticus* caused macroscopic and histological confluent pneumonia with significant increase in BAL white cell count, tumor necrosis factor-α (TNF-α), and macrophage inflammatory protein (MIP)-2. At 12 h, high concentrations of *S. haemolyticus* were identified in BAL. At 36 h, lung injury and BAL inflammation were less severe than at 12 h and moderate concentrations of species belonging to the oropharyngeal flora were identified in lung tissue. The inoculation of 1.0 × 10^6^ and 1.0 × 10^7^ CFU live *S. haemolyticus* caused histologic interstitial pneumonia and moderate BAL inflammation. Similar results were observed after inoculation of inactivated *S. haemolyticus*. Moreover, biofilm formation was a common phenotype in *S. haemolyticus* isolates. The low prevalence of the *ica* operon in our clinical *S. haemolyticus* strain collection indicated *icaA* and *icaD* independent-biofilm formation.

**Conclusion:**

In immunocompetent spontaneously breathing mice, inoculation of *S. haemolyticus* causes concentration-dependent lung infection that spontaneously recovers over time. *icaA* and *icaD* independent biofilm formation is a common phenotype in *S. haemolyticus* isolates.

## Introduction

Coagulase-negative staphylococci (CoNS), ubiquitous colonizers of healthy skin and mucosal surfaces, are among the most frequently isolated bacteria in the microbiology laboratory. Initially considered as non-pathogenic, CoNS has been recognized over the past three decades as nosocomial pathogens causing significant morbidity, mortality, and healthcare costs worldwide (Becker et al., 2014), in adults (Wisplinghoff et al., 2004; Rogers et al., 2009) and newborns (Cunha Mde et al., 2002; Venkatesh et al., 2006; Rogers et al., 2009). Because of their propensity to form biofilms on device materials (John and Harvin, 2007), they frequently infect catheters inserted in critically ill patients (Rogers et al., 2009; Becker et al., 2014; Grasselli et al., 2017) and prosthesis implemented during orthopedic surgery (Wisplinghoff et al., 2004; Becker et al., 2014). CoNS have also been increasingly reported as a cause of bacteremia in immunocompromised and hospitalized patients, especially with indwelling medical devices (Bearman and Wenzel, 2005; Cervera et al., 2009; Rogers et al., 2009; Becker et al., 2014). Although frequently retrieved in tracheal secretions (Agvald-Ohman et al., 2004; Qi et al., 2015), some controversy remains whether CoNS can infect lung parenchyma, particularly in critically ill patients requiring prolonged mechanical ventilation through endotracheal tube or tracheostomy.

The molecular basis of the virulence of CoNS remains largely unknown. It has been demonstrated that the formation of biofilm is responsible for the indolent, difficult-to-treat nature of CoNS infections, but the phenotype may vary among different species. *Staphylococcus epidermidis* (*S. epidermidis*) requires a functional *ica* locus (Loiez et al., 2007; Peer et al., 2011; Morfin-Otero et al., 2012) when producing a thick biofilm consisting mainly of polysaccharide intercellular adhesion (PIA) (von Eiff et al., 2005), thus creating an environment that results in antibiotic tolerance and evasion of host defense (Crass and Bergdoll, 1986; Lina et al., 1996). In contrast to *S. epidermidis*, proteins and extracellular DNA are of more functional relevance for biofilm accumulation in *Staphylococcus haemolyticus* (*S. haemolyticus*), whereas PIA plays only a minor role (Fredheim et al., 2009). Whether the phenotype of biofilm formation, especially *ica* locus, is critical for the virulence of CoNS remains unclear.

The aim of this study was to assess whether CoNS can infect normal lung parenchyma; we set up an experimental model in mice in which increasing concentrations from 10^6^ to 10^8^ colony forming units (CFU) of *S. haemolyticus* strains were inoculated in the trachea. In addition, the phenotype of biofilm formation in *S. haemolyticus* was investigated.

## Materials and Methods

### Strain Identification and Preparation

**FIGURE 1:**
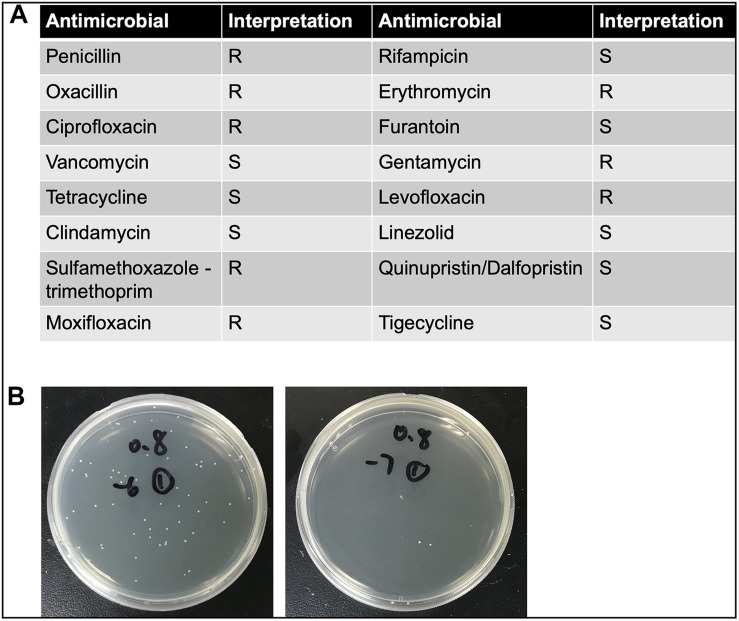
**(A)** The detailed antimicrobial resistance profile of *S. haemolyticus* isolated from the blood culture specimen of a female hospitalized patient with Hodgkin’s lymphoma. **(B)** The number of colony forming units (CFU) was calculated when optical density value was 0.8. The left panel showed 10^–6^ times diluted inoculum, and the right panel showed 10^–7^ times diluted inoculum.

The clinical CoNS strain, obtained from the Ruijin Hospital, Shanghai Jiao Tong University School of Medicine, was isolated from the blood culture specimen of a female hospitalized patient with Hodgkin’s lymphoma. The strain was identified as *S. haemolyticus* by matrix-assisted laser desorption/ionization time of flight mass spectrometry (MALDI-TOF-MS) (Biomerieux VITEK MSTM, France). The isolate was incubated overnight in Luria-Bertani (LB) broth, shaken at 37°C at the rate of 275 rpm. One hundred microliters of inoculum was pipetted into 5 ml of fresh LB broth, shaken at 37°C at the same rate, for approximately 2 h, with additional adjustment (either re-shaking or diluting) if needed, until optical density (OD) value (600 nm) reached 0.8. Number of CFU was previously calculated: OD_600__nm_ = 0.8 corresponds to 5 × 10^8^ CFU/ml (Figure 1B). The detailed antimicrobial resistance profile is shown in Figure 1A. To prepare the different bacterial concentrations, the adjusted inocula were centrifuged at 10,000 × *g* for 2 min and pellets were subsequently re-suspended in phosphate-buffered saline (PBS) with the final concentration of 2.5 × 10^7^, 2.5 × 10^8^, and 2.5 × 10^9^ CFU/ml. In separate experiments, inactivated inocula were pretreated by incubating at 65°C for 30 min after centrifugation.

### Experimental Model and Measurements

C57BL/6 male mice (7–8 weeks) were obtained from Shanghai Laboratory Animal Co., Ltd., China. Animals were kept in a specific pathogen-free facility of the Research Center for Experimental Medicine of Ruijin Hospital, Shanghai Jiao Tong University School of Medicine, China. All animal procedures were approved by the Ruijin Hospital Animal Ethics Committee. Mice were anesthetized with pentobarbital sodium salt (40 mg/kg) intraperitoneally and received intratracheally: (1) PBS as carrier control; and (2) 40 μl of *S. haemolyticus* in concentrations of 2.5 × 10^7^, 2.5 × 10^8^, and 2.5 × 10^9^ CFU/ml (equivalent to inocula of 1 × 10^6^, 1 × 10^7^, and 1 × 10^8^ CFU, respectively), according to protocols previously reported (Su et al., 2004). In separate experiments, we inoculated with 40 μl of inactivated *S. haemolyticus* in concentrations of 2.5 × 10^7^, 2.5 × 10^8^, and 2.5 × 10^9^ CFU/ml. After 12 or 36 h, both bronchoalveolar lavage (BAL) samples and lungs were collected following sacrifice.

### Measurement of Neutrophil Counts, Cytokines, and Protein Levels in BAL Fluid

Total cell count and differential in BAL were obtained using Sysmex pocH-100i (Sysmex Shanghai Ltd., China) according to the manufacturer’s protocols. BAL samples were centrifuged at 3000 rpm for 10 min, and the supernatants were collected and stored at −20°C. Macrophage inflammatory protein (MIP)-2, a mouse neutrophil chemokine, as well as tumor necrosis factor-α (TNF-α) were measured by ELISA (R&D Systems, Minneapolis, MN, United States) in the BAL supernatants. BAL protein concentration as a marker of lung endothelial and epithelial permeability was also measured using Bthe CA Protein Assay Kit^1^ (Beyotime, Shanghai, China).

### CoNS Quantification in BAL Fluid and Lung Homogenate

**FIGURE 2:**
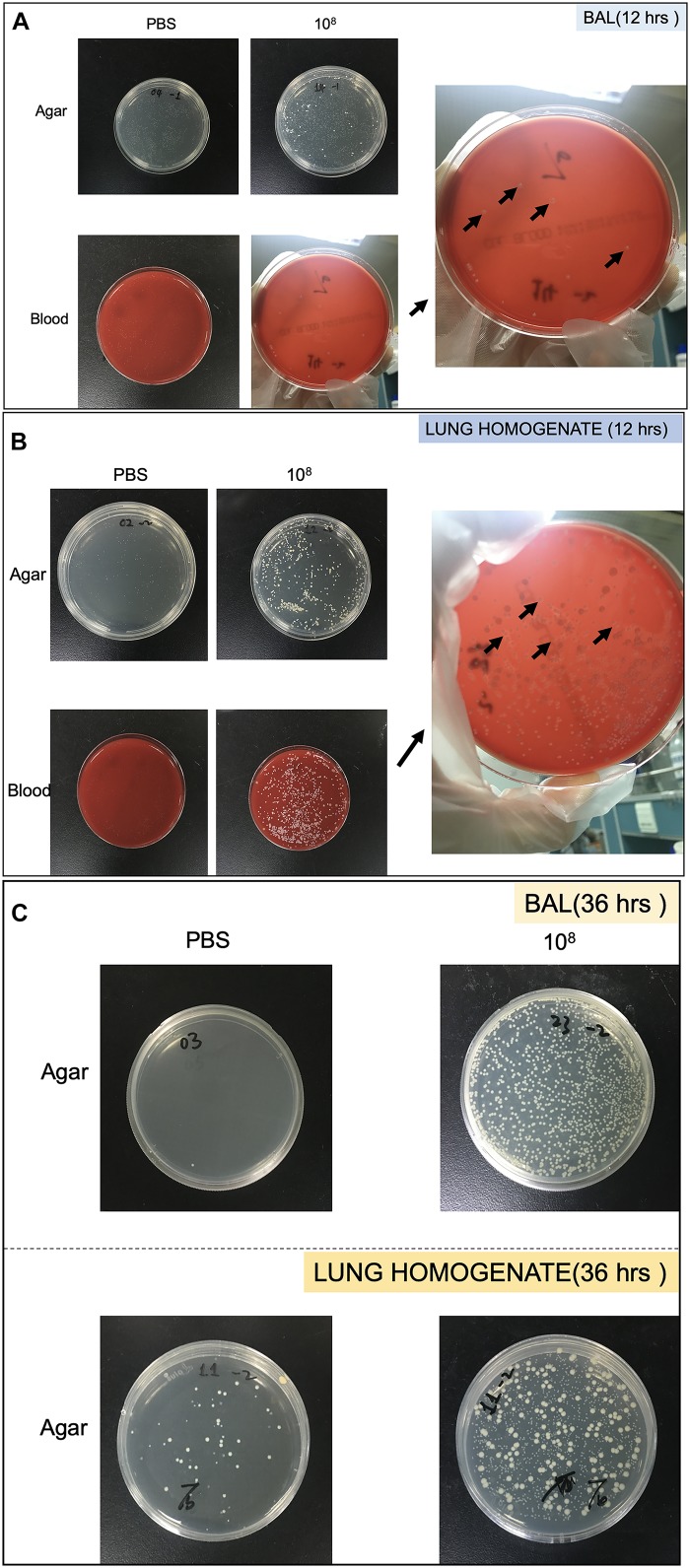
Pictures of Petri dish showing the heterogeneity of strains growth in bronchoalveolar lavage (BAL) and lung homogenate. **(A)** Strains collected from BAL at 12 h. **(B)** Strains collected from lung homogenate at 12 h. **(C)** Strains collected from BAL and lung homogenate at 36 h. The black arrows showed colonies identified as *S. haemolyticus*.

Bacterial growth in the BAL and lung homogenate following CoNS-induced lung injury *in vivo* were quantitated by CFU. The samples were cultured on LB agar plate (TEKnova, Hollister, CA, United States) as well as blood plate (CP Adaltis Diagnostics, Shanghai, China) overnight at 37°C. Individuals colonies (CFU) were then counted and also identified as CoNS by MALDI-TOF-MS (Figures 2A–C).

### Histology

After excision, lungs were gently inflated with 0.5 ml of 4% formalin, followed by tracheal ligature, fixed in 4% formalin, and dehydrated through serially diluted graded ethyl alcohol baths. After fixation, lungs were embedded in paraffin, cut into 5-μm sections, and stained with hematoxylin and eosin. An independent investigator, blinded to group assignments, assessed whether lung parenchyma was infected or not. Pneumonia was defined as the presence of polymorphonuclear neutrophils (PMNs) within septa and alveolar spaces (Mizgerd and Skerrett, 2008). Levels of lung injury were assessed using the lung injury score (LIS) (Matute-Bello et al., 2011), which varies from 0 to 1, where a higher score means more injury (Supplementary Table S1).

### Biofilm-Associated Genes Detection

Genomic DNA isolation from the *S. haemolyticus* strain used in our experiment as well as 19 other clinical *S. haemolyticus* strains were carried out as previously described (Frebourg et al., 2000; Zhou et al., 2019). Standard strains of *S. haemolyticus* (ATCC29970) and *S. epidermidis* (ATCC12228) were used as controls. The presence of *icaA* and *icaD* genes (main component of PIA biofilm) along with a variety of significant biofilm-associated genes [encoding cell surface attachment proteins (*ebpS, cbp, fbp*, and *srtA*), quorum sensing (*agrA, agrB, agrD*, and *luxS*), and eDNA release (*cidA, cidB, lrgA, lrgB*, and *atlE*)] were detected in all the *S. haemolyticus* strains by polymerase chain reaction (PCR). Amplification conditions (20 μl volume) were as follows: denaturation for 3 min at 94°C, followed by 30 cycles of 94°C for 30 s, 55°C for 30 s, and 72°C for 1 min, with final extension at 72°C for 5 min. The primer sequences of PCR used in this study are shown in Supplementary Table S2 (Panda and Singh, 2018). The amplified PCR products were analyzed on 1.5% agarose gel and were visualized using a UV trans-illuminator^2^ (FR980A, Furi Technology, China). In addition, the PCR products were sequenced to confirm the product identity.

### Detection of Biofilm Production by Microtiter Plate Assay

The capacity of biofilm formed by *S. haemolyticus* was determined by a modified method (Fredheim et al., 2009; Panda and Singh, 2018). Bacterial suspension was prepared in lysogeny broth (LB) and adjusted to 0.5 McFarland (1 × 10^8^ CFU/ml). This bacterial suspension was 20-fold (1/20) diluted to reach 5 × 10^6^ CFU/ml. Then, 180 μl of LB and 20 μl of bacterial suspensions were inoculated into 96-well flat-bottomed sterile polystyrene microplate. Microplates were incubated at 37°C for 24 h and then were washed twice by PBS. Biofilms formed on the walls of microplate were fixed by adding 150 μl of methanol and then stained with 150 μl of crystal violet (Beyotime, Shanghai, China) for 15 min. Then crystal violet-stained microplates were washed twice with PBS to discharge crystal violet stain. The microplate was measured spectrophotometrically at 570 nm by a microplate reader. Isolates were considered biofilm positive if they had an absorbance ≥0.12. We performed the assay in a single run of four wells in biological triplicates.

### Statistical Analysis

Results are expressed as median and 25th–75th interquartile range (IQR). Comparisons between two groups were made by non-parametric Mann–Whitney *U* test. Comparisons between more than two groups were made by non-parametric Kruskal–Wallis analysis followed by pairwise comparisons tested using a *post hoc* analysis Dunn’s method. A *p*-value < 0.05 was considered statistically significant. All statistical analysis was performed using GraphPad Prism software (La Jolla, CA, United States).

## Results

### Groups and Mice Behavior Following *S. haemolyticus* Tracheal Inoculation

As shown in Table 1, 221 mice were distributed in 33 groups. Each animal was anesthetized, inoculated, sent back to its cage, and sacrificed at 12 or 36 h.

**TABLE 1:** Groups of mice in the study.

		**Mice for lung histology (lung injury score)**	**Mice for BAL analysis (white cells, TNFα, MIP-2, proteins, BAL quantitative bacteriology after live CoNS tracheal inoculation)**	**Mice for quantitative lung tissue bacteriology**
**Eighteen groups of mice sacrificed at H36 *n* = 178**				
PBS		6	13	13
CoNS 10^6^	Inactivated	6	6	–
	Live	6	17	9
CoNS 10^7^	Inactivated	6	7	–
	Live	6	28	10
CoNS 10^8^	Inactivated	6	6	–
	Live	6	17	10
**Eight groups of mice sacrificed at H12 *n* = 43**				
PBS		6	5	–
CoNS 10^6^	Live	5	6	–
CoNS 10^7^	Live	5	5	–
CoNS 10^8^	Live	6	5	–

*PBS, phosphate-buffered saline; BAL, bronchoalveolar lavage; CoNS, inoculation of coagulase negative *Staphylococcus haemolyticus*, whose inoculum is expressed in colony forming units; H36 = 36 h; H12 = 12 h.*

As shown in the Supplementary Videos available on the online data supplement, mice behavior was deeply affected only in animals inoculated with 10^8^ CFU live *S. haemolyticus*. Like in other models of inoculation pneumonia, mice cage physical activity and behavior were affected (reduced mobility and lack of food intake) only transitorily during the first 12 h. Subsequently, they progressively recovered, and all animals survived at 36 h.

### Systemic Inflammation Following *S. haemolyticus* Tracheal Inoculation

**FIGURE 3:**
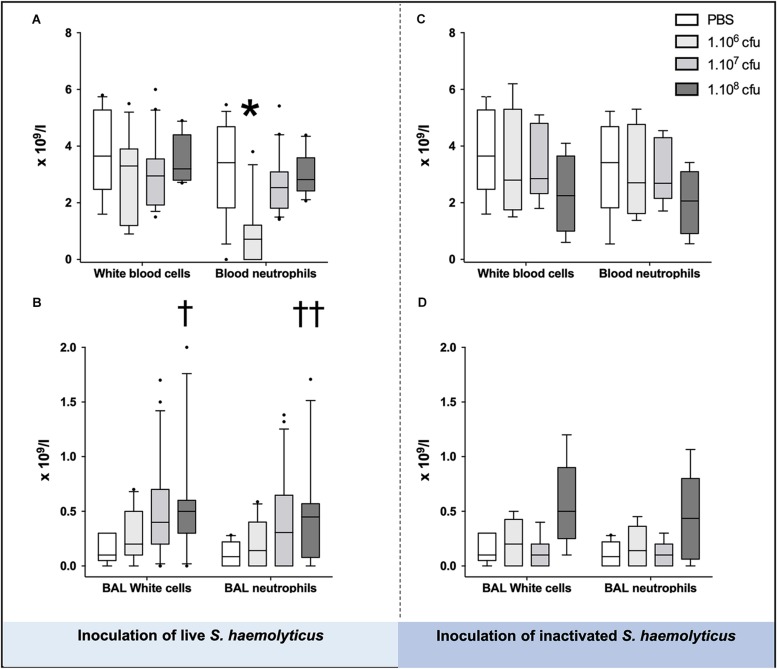
White cells and neutrophils in the blood **(A,C)** and BAL **(B,D)**, 36 h following tracheal inoculation of phosphate-buffered saline (PBS), live and inactivated *S. haemolyticus*. Median values, interquartile 25th–75th range and extreme values (>2 SD) are represented. Blood neutrophils were significantly different between groups: Kruskal–Wallis *p* = 0.0095; ^∗^ indicates *p* < 0.05 for 10^6^ CFU versus PBS and 10^7^ CFU; BAL White cells were significantly different between groups: Kruskal–Wallis *p* = 0.0023; ^†^indicates *p* = 0.0013 for 10^8^ CFU versus PBS; BAL neutrophils were significantly different between groups: Kruskal–Wallis *p* = 0.01; ^††^indicates *p* = 0.0084 for 10^8^ CFU versus PBS.

As shown in Figures 3A,C, blood neutrophil counts significantly decreased in animals inoculated with 10^6^ CFU as compared with PBS-treated mice (1.538 ± 1.661 for 10^6^ CFU, 3.138 ± 1.645 for PBS, *p* = 0.02 for 10^6^ CFU vs. PBS) or 10^7^ CFU treated mice (1.538 ± 1.661 for 10^6^ CFU, 2.783 ± 1.101 for 10^7^ CFU, *p* = 0.03 for 10^6^ CFU vs. 10^7^ CFU), but did not change in animals inoculated with 10^8^ CFU live or inactivated *S. haemolyticus* whatever the inoculum.

### *S. haemolyticus*-Induced Lung Histological Damage

**FIGURE 4:**
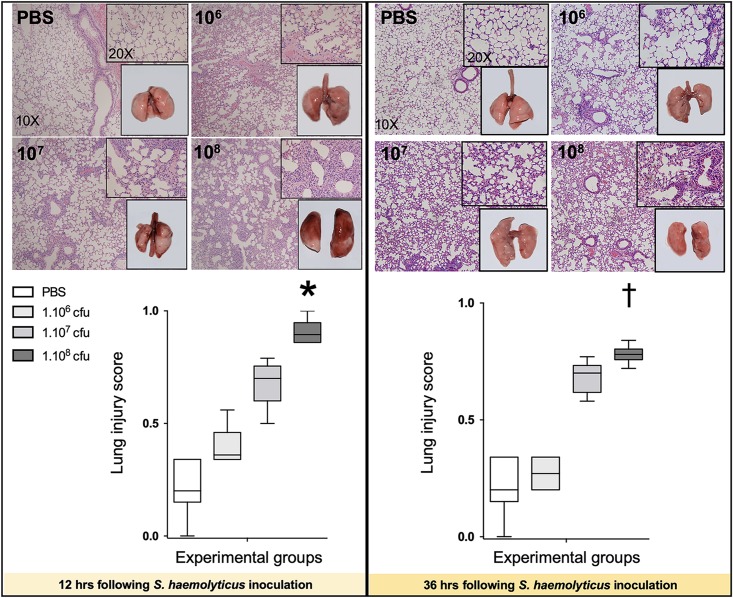
Macroscopic (square windows) and histological lung aspects 12 and 36 h following tracheal inoculation of PBS, and live *S. haemolyticus* at 10^6^, 10^7^, and 10^8^ CFU. Lung injury score (LIS) is represented as median values and interquartile 25th–75th range and extreme values (>2 SD). Twelve and 36 h after inoculation, LIS was significantly different between groups: Kruskal–Wallis *p* = 0.0002; ^∗^indicates *p* < 0.05 for 10^8^ CFU versus PBS and 10^6^ CFU; ^†^indicates *p* < 0.05 for 10^8^ CFU versus PBS and 10^6^ CFU. Twelve and 36 h after tracheal inoculation of 10^6^ and 10^7^ CFU, interstitial pneumonia is present, characterized by inflammatory cells infiltrating interalveolar septa and respecting alveolar space and lung architecture. Twelve and 36 h after tracheal inoculation of 10^8^ CFU, confluent pneumonia is present, characterized by inflammatory cells invading alveolar space, hyaline membranes, lung consolidation, and loss of basal lung architecture.

As shown in Figure 4, 12 and 36 h following inoculation of 10^8^ CFU live *S. haemolyticus*, there was macroscopic evidence of pneumonia. Twelve hours after PBS inoculation, macroscopic injury was absent. Macroscopic injury was moderate 12 h after inoculation of 10^6^ and 10^7^ CFU live *S. haemolyticus* and severe 12 h after inoculation of 10^8^ CFU live *S. haemolyticus*. Thirty-six hours later, macroscopic injury was less pronounced, corresponding to spontaneous improvement in behavior. Histologically, interstitial pneumonia was observed after inoculation of 10^6^ and 10^7^ CFU live *S. haemolyticus* and confluent pneumonia was observed after inoculation of 10^8^ CFU live *S. haemolyticus*. At 12 h, LIS increased linearly with the tracheal inoculum (*p* < 0.001). At 36 h, LIS of animals inoculated with 10^6^ CFU live *S. haemolyticus* returned to control values. LIS of animals inoculated with 10^7^ CFU live *S. haemolyticus* remained elevated whereas LIS of animals inoculated with 10^8^ CFU live *S. haemolyticus* also remained elevated, but significantly lower than after 12 h (0.780 [0.757–0.802] at 36 h versus 0.895 [0.860–0.947] at 12 h, *p* = 0.002).

**FIGURE 5:**
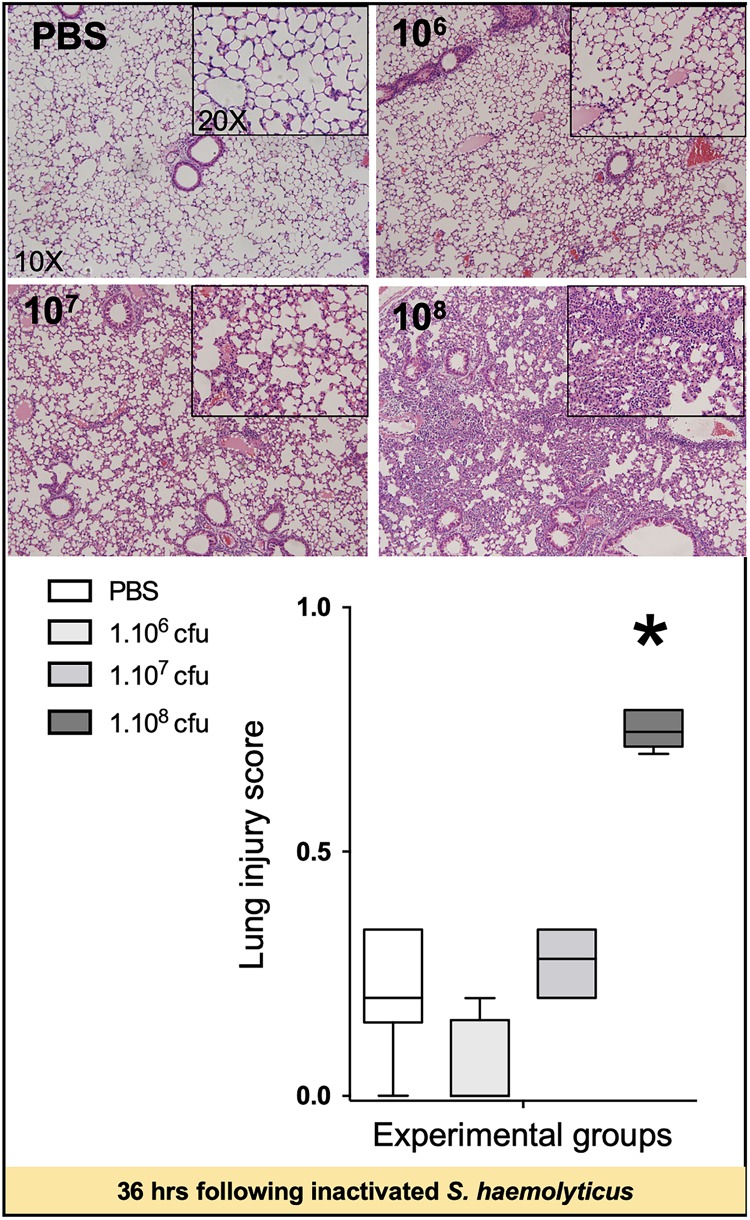
Lung histology 36 h following tracheal inoculation of PBS and inactivated *S. haemolyticus* at increasing load 10^6^, 10^7^, and 10^8^ CFU. LIS is represented as median values and interquartile 25th–75th range and extreme values (>2 SD). LIS is significantly different between groups: Kruskal–Wallis *p* = 0.0004; ^∗^indicates *p* < 0.05 for 10^8^ CFU versus PBS and 10^6^ CFU. Thirty-six hours after tracheal inoculation of inactivated *S. haemolyticus* at 10^6^ and 10^7^ CFU, inflammatory cells infiltrate interalveolar septae, respecting alveolar space and lung architecture. The inoculation of 10^8^ CFU of inactivated *S. haemolyticus* induced invasion of inflammatory cells within alveolar space, hyaline membrane formation, and lung consolidation.

As shown in Figure 5, tracheal inoculation of 10^8^ CFU inactivated *S. haemolyticus* induced severe lung lesions at 36 h, whereas less histological injury was observed 36 h following the inoculation of 10^6^ or 10^7^ CFU inactivated *S. haemolyticus*.

### Lung Inflammation and Protein Permeability Following *S. haemolyticus* Tracheal Inoculation

**FIGURE 6:**
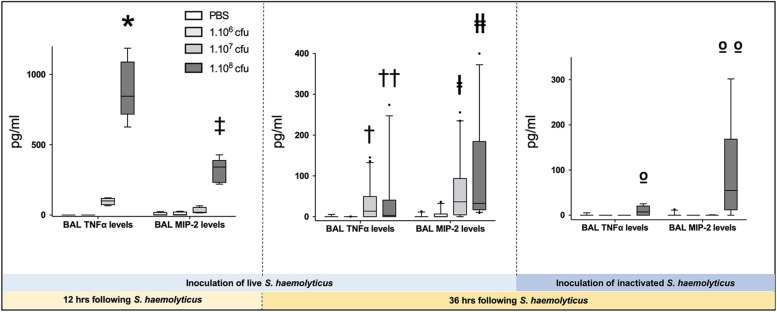
Tumor necrosis factor (TNF-α) and monocyte inflammatory protein-2 (MIP-2) in BAL following tracheal inoculation of PBS, and increasing inoculum (10^6^, 10^7^ and 10^8^ CFU) of live and inactivated *S. haemolyticus.* Data are expressed as median values, interquartile 25th–75th range and extreme values (>2 SD). Twelve hours following tracheal inoculation of live *S. haemolyticus*, alveolar levels of TNF-α and MIP-2 significantly increased with the inoculum (Kruskal–Wallis *p* = 0.0002 for TNF-α and *p* = 0.004 for MIP-2; ^∗^ and ‡ indicate *p* < 0.05 for 10^8^ CFU versus PBS and 10^6^ CFU). Thirty-six hours following inoculation, alveolar levels of TNF-α and MIP-2 remained significantly elevated in mice that received the 10^8^ CFU (Kruskal–Wallis *p* = 0.0012 for TNF-α and *p* < 10^–4^ for MIP-2; ^††^ and ╪╪ indicate *p* < 0.05 for 10^8^ CFU versus PBS and 10^6^ CFU; ^†^indicates *p* = 0.03 for 10^7^ versus 10^6^ CFU and ╪ indicates *p* < 0.05 for 10^7^ versus PBS and 10^6^ CFU). However, both cytokine levels were significantly lower than levels measured 12 h following inoculation of 10^8^ CFU *S. haemolyticus* (*p* < 10^–4^ for TNF-α and MIP2 alveolar levels at 12 h versus 36 h). Thirty-six hours following tracheal inoculation of 10^8^ CFU inactivated *S. haemolyticus*, BAL TNF-α and MIP-2 significantly increased with the inoculum (Kruskal–Wallis *p* = 0.0086 for TNF-α and *p* = 0.0013 for MIP-2; ^ο^ indicate *p* < 0.05 for 10^8^ versus 10^7^ and 10^6^ CFU; ^οο^ indicate *p* < 0.05 for 10^8^ versus 10^7^ and 10^6^ CFU, and PBS).

As shown in Figure 3B, tracheal inoculation of 10^8^ CFU live *S. haemolyticus* produced a significant increase in BAL influx of white cells, specifically neutrophils (*p* = 0.0013 for 10^8^ CFU versus PBS for white cells and *p* = 0.0084 for 10^8^ CFU versus PBS for neutrophils). As shown in Figure 3D, tracheal inoculation of increasing inocula of inactivated *S. haemolyticus* did not produce any significant increase of BAL white cells and neutrophils. As shown in Figure 6, high peaks of TNF-α and MIP-2 were observed in BAL 12 h following inoculation of live *S. haemolyticus*: TNF-α was (median, [25th–75th] percentile) 845.2 [717.8–1088] pg/ml in mice that received a 10^8^ CFU inoculum and 0 [0–0] in mice that received PBS (*p* = 0.0021) and 0 [0–0] in mice that received a 10^6^ inoculum (*p* = 0.0011); MIP-2 was 341.8 [232.9–388.2] pg/ml in mice that received a 10^8^ CFU inoculum, 2.2 [0.1–18.5] in mice that received PBS (*p* = 0.0094), and 3.0 [0–22] in mice that received a 10^6^ inoculum (*p* = 0.0084). Thirty-six hours following inoculation of live *S. haemolyticus*, TNF-α was (median, [25th–75th] percentile) 4.6 [0–22] pg/ml in mice that received a 10^8^ CFU inoculum, 0 [0–0] in mice that received PBS (*p* = 0.047), and 0 [0–0] in mice that received a 10^6^ inoculum (*p* = 0.004); TNF-α was significantly higher in mice that received a 10^7^ inoculum compared to those that received a 10^6^ inoculum: 0 [0–29.3] vs. 0 [0–0] (*p* = 0.03); MIP-2 was 45.7 [16.5–154] pg/ml in mice that received a 10^8^ CFU inoculum, 0 [0–0] in mice that received PBS (*p* < 10^–4^), and 0 [0–1.1] in mice receiving 10^6^ inoculum (*p* < 10^–4^); MIP-2 was significantly higher in mice that received a 10^7^ inoculum compared to mice that received either PBS or a 10^6^ inoculum: 13.2 [0–80.3] versus 0 [0–0], (*p* = 0.03), and 13.2 [0–80.3] versus 0 [0–1.1], (*p* = 0.04), respectively. Thirty-six hours following inoculation of inactivated *S. haemolyticus*, TNF-α was 7.3 [0–20.4] pg/ml in mice that received a 10^8^ CFU inoculum, 0 [0–0] in mice that received PBS (*p* = 0.07), 0 [0–0] in mice that received a 10^6^ inoculum (*p* = 0.02), and 0 [0–0] pg/ml in mice that received a 10^7^ CFU inoculum (*p* = 0.01); MIP-2 was 54.7 [12.1–168.2] pg/ml in mice that received a 10^8^ CFU inoculum, 0 [0–0] in mice that received PBS (*p* = 0.0064), 0 [0–0] in mice that received a 10^6^ inoculum (*p* = 0.0031), and 0 [0–0] pg/ml in mice that received a 10^7^ CFU inoculum (*p* = 0.0090).

**FIGURE 7:**
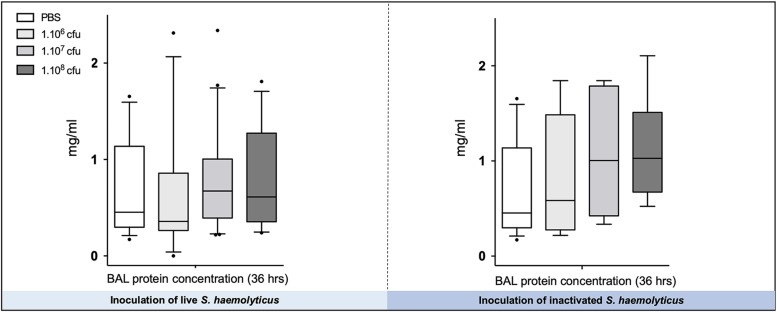
The total protein concentration in BAL following tracheal inoculation of PBS, and increasing inocula (10^6^, 10^7^, and 10^8^ CFU) of live and inactivated *S. haemolyticus* remained unchanged. Data are expressed as median values, interquartile 25th–75th range, and extreme values (>2 SD).

As shown in Figure 7, there were no significant differences in the total protein concentration in BAL following tracheal inoculation of PBS or 10^6^, 10^7^, and 10^8^ CFU of live and inactivated *S. haemolyticus.*

### Lung Tissue Bacterial Load Following *S. haemolyticus* Tracheal Inoculation

**FIGURE 8:**
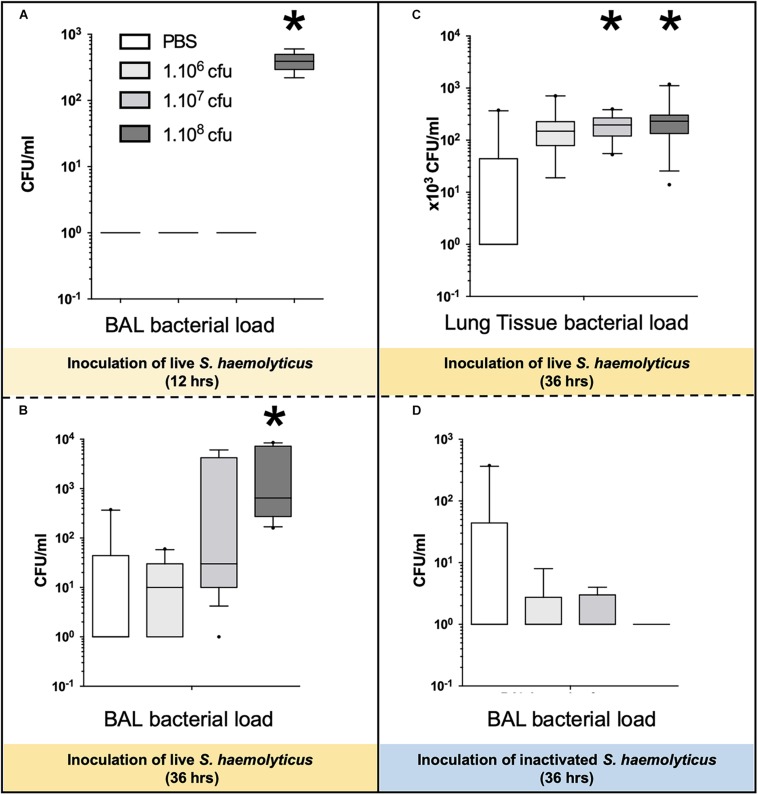
Bacterial load in BAL and lung tissue homogenate following tracheal inoculation of PBS, and increasing inocula (10^6^, 10^7^, and 10^8^ CFU) of live and inactivated *S. haemolyticus.* Data are expressed as median values, interquartile 25th–75th range and extreme values (>2 SD). **(A)** Kruskal–Wallis *p* = 0.002; ^∗^indicates *p* < 0.05 for 10^8^ CFU versus PBS and 10^6^ CFU; **(B)** Kruskal–Wallis *p* = 0.04; ^∗^indicates *p* = 0.046 for 10^8^ CFU versus 10^6^ CFU; **(C)** Kruskal–Wallis *p* = 0.003; ^∗^indicates *p* < 0.05 for 10^8^ CFU and 10^7^ CFU versus PBS. **(D)** No significant differences of bacterial colonies in BALF among the inactivated *S. haemolyticus* groups.

As shown in Figure 8A, 12 h following the tracheal inoculation of 10^8^ CFU *S. haemolyticus*, bacterial load in the BAL reached a median value of 390 CFU/ml [294 to 496], whereas BAL from the other groups remained sterile. At the 12-h time point, the overall identified strains in both BAL (Figure 2A) and lung homogenate (Figure 2B) samples were *S. haemolyticus*. As shown in Figure 8B, 36 h later, bacterial load remained elevated after inoculation of 10^8^ CFU *S. haemolyticus* and appeared in BAL following PBS and lower inocula. As shown in Figure 8C, a significant bacterial load was observed in lung tissue homogenates 36 h following tracheal inoculation of 10^7^ and 10^8^ CFU *S. haemolyticus* (*p* = 0.003). However, colonies in both BAL and lung homogenate (Figure 2C) were identified as species originating from oropharyngeal flora, such as *Achromobacter denitrificans* and *Bordetella avium*. *S. haemolyticus* strains remained undetectable. As shown in Figure 8D, 36 h following tracheal inoculation of PBS and increasing inocula of inactivated *S. haemolyticus*, a weak and non-significant bacterial load was observed in BAL.

### Biofilm Formation of CoNS Strains

**FIGURE 9:**
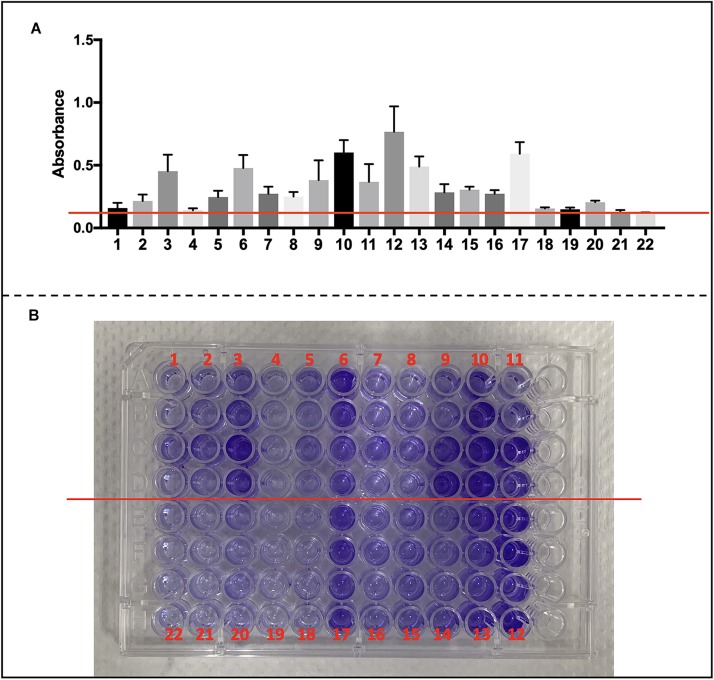
Biofilm formation of CoNS strains and detection of biofilm-associated genes. **(A)** Data are shown in mean ± SD. Mean level of absorbance of wells was measured spectrophotometrically at 570 nm by a microplate reader after staining by crystal violet (red cross line shows cutoff value = 0.12). **(B)** Images of wells represent each strain in crystal violet-stained microplates.

We collected *S. haemolyticus* used in our *vivo* experiment as well as another 19 of clinical *S. haemolyticus* strains and screened for biofilm-associated genes. The details of origin and antimicrobial resistance profile are listed in Supplementary Tables S3, S4. All the *S. haemolyticus* isolates formed a biofilm on microplates (Figures 9A,B).

As shown in Supplementary Figure S1A, none of the *S. haemolyticus* carried *icaA* or *icaD* genes. As shown in Supplementary Figure S1B, 14 isolates (14/20, 70%) of clinical *S. haemolyticus* carried the *cbp* gene while 18 isolates (18/20, 90%) carried the *argD* gene. All the clinical *S. haemolyticus* isolates carried the genes encoding cell surface attachment proteins (*ebpS*, *fbp*, and *srtA*), quorum sensing (*agrA*, *agrB*, and *luxS*), and eDNA release (*cidA*, *cidB*, *lrgA*, *lrgB*, and *atlE*) (Supplementary Figures S1C–H).

## Discussion

The main findings of this work can be summarized as follows. (1) Twelve hours after the tracheal inoculation of 10^8^ CFU live *S. haemolyticus*, macroscopic evidence of lung injury was observed, histologically characterized by confluent pneumonia and associated with marked increases in TNF-α and MIP-2 and the presence of significant concentrations of *S. haemolyticus* in the BAL and in the lung homogenate. (2) Thirty-six hours later, macroscopic and histologic evidence of confluent pneumonia and BAL inflammation persisted, but at a lesser degree. High concentrations of bacteria were found in lung tissue and BAL, but *S. haemolyticus* strains were replaced by various species belonging to the oropharyngeal flora. (3) Tracheal inoculation of lower concentrations of live *S. haemolyticus* produced less severe macroscopic lung injury, histological evidence of interstitial pneumonia, and moderate but significant inflammation in BAL. (4) Inoculation of increasing concentrations of inactivated *S. haemolyticus* produced similar histological lung injury with low-to-moderate alveolar inflammation, which were similar to those found 36 h following tracheal inoculation of 10^8^ CFU live *S. haemolyticus*. (5) Biofilm formation was a common phenotype in *S. haemolyticus* isolates. The low prevalence of the *ica* operon in our clinical *S. haemolyticus* strain collection indicated *icaA* and *icaD* independent-biofilm formation.

CoNS are considered to be among the top common pathogens isolated in bloodstream infections (Moyo et al., 2010; Adam et al., 2011; Wilson et al., 2011). The *S. haemolyticus* isolate inoculated in our mice was obtained from a patient with nosocomial bacteremia and considered by clinicians as pathogenic, requiring antibiotic treatment. The presence of CoNS in distal bronchial specimens is generally believed to reflect lung colonization or contamination (van Saene et al., 1996). However, more and more physicians consider that CoNS can generate pulmonary infections in both immunocompetent and immunocompromised hosts (Rouby et al., 1992; Kloos and Bannerman, 1994; Kirtland et al., 1997; Rello et al., 1997; George et al., 1998; Hell et al., 1999). Staphylococci of the *S. epidermidis* group demonstrate a low to moderate virulence (Becker et al., 2014). They have a high ability to attach to foreign bodies’ surface and, sometimes, host cells. *S. haemolyticus* is able to induce biofilm on cultured cells (Shrestha et al., 2018) and *Staphylococcus lugdunensis* may adhere and invade cultured alveolar type II cells (Pereira et al., 2012). Formed on foreign bodies and endocardium cells, biofilm makes the cells less accessible to the defense system of the host, impairs antibiotic activity, and is the most important virulence factor for CoNS (Becker et al., 2014). However, the production of strongly cytotoxic phenol-soluble modulins by CoNS is low, whereas the release of pyrogenic toxin super-antigen like staphylococcal enterotoxins and toxic shock syndrome toxin1 is virtually absent. Cell wall components of *S. epidermidis* stimulate the release of TNF-α, IL-6, and IL-1β from activated monocytes (Mattsson et al., 1993, 1996). No data are available concerning the pathogenicity of *S. haemolyticus* on the lungs. Our study demonstrates for the first time that intratracheal inoculation of *S. haemolyticus* induces histological interstitial and confluent pneumonia in immunocompetent mice with influx of inflammatory cells and cytokines in BAL. Twelve hours after a bacterial inoculation of 10^8^ CFU, low to moderate concentrations of *S. haemolyticus* were retrieved in the BAL. Twenty-four hours later, while we attempted to select as many colonies as possible with different morphologies (appearance, size, shape), we were unsuccessful in detecting *S. haemolyticus*, either massively diluted into colonizing strains, or cleared by the immune systems at those time points. Species such as *A. denitrificans* and *B. avium*, belonging to the oropharyngeal flora, were isolated at low concentrations in lung parenchyma. All these results tend to indicate that the *S. haemolyticus* colonies initially inoculated, and found at the 12-h time point, were eventually replaced by germs colonizing the airways between the 12- and 36-h time point. Supporting this hypothesis, BAL TNF-α and MIP-2 were quite elevated 12 h after the inoculation of 10^8^ CFU *S. haemolyticus* and markedly decreased 24 h later, suggesting a decreased monocyte activation by wall components of the inoculated isolates. All these data confirmed the hypothesis of *S. haemolyticus* pathogenicity to cause pneumonia, from which immunocompetent mice are able to recover spontaneously.

We previously reported that pneumonia could be induced by tracheal inoculation of endotoxin (Zhu et al., 2014; Tang et al., 2017), 10^6^ CFU *Pseudomonas aeruginosa* (Mao et al., 2015) and *Escherichia coli* (Monsel et al., 2015) in the same mice model. In the current study, 10^7^ CFU inoculum was required to produce interstitial pneumonia and 10^8^ CFU was required to produce confluent pneumonia, confirming indirectly that *S. haemolyticus* is less virulent than Gram-negative bacteria (van Saene et al., 1996; Lambotte et al., 2002; Becker et al., 2014). Interestingly, similar inflammatory cell influx, release of proinflammatory cytokines in the BAL, and lung parenchymal histological damage were also produced by high inoculum of inactivated *S. haemolyticus*, suggesting that wall debris may participate in lung injury. Another interesting finding is that inoculation pneumonia only transitorily impacted mice behavior. Animals inoculated by 10^8^ CFU *S. haemolyticus* were initially prostrated with limited food access, but rapidly recovered, moving again within their cage and accessing food and water. Although histological pneumonia persisted, this behavior improvement was associated with a decreased lung inflammation and a partial eradication of *S. haemolyticus* from the lung.

It has been described that biofilm formation was a two-step process that was mediated by a number of factors like cell wall-anchored surface proteins (i.e., *fbe* and *bhp*) (Cucarella et al., 2001) and the cell wall lytic enzyme autolysin E (i.e., *atlE*) (Heilmann et al., 1997; Davis et al., 2001). *S. haemolyticus* biofilm formation *in vitro* has been reported, but the contribution of *ica* operon was not well elucidated (Cramton et al., 1999; Allignet et al., 2001; Foka et al., 2006; Frank and Patel, 2007). In our study, we have investigated the biofilm formation phenotypes and genotypes of a large collection of clinical *S. haemolyticus* isolates, and the genetic background of our *vivo* experiment strain for biofilm formation is mostly similar as what is commonly found in *S. haemolyticus* strains from other studies (Fredheim et al., 2009). We confirmed by sequencing that the *icaA* and *icaD* was absent in all the *S. haemolyticus* isolates, suggesting a low prevalence compared with *S. epidermidis*. In concurrence with previously reported sequences (Takeuchi et al., 2005; Watanabe et al., 2007), the instability of the *ica* operon may be explained by genomic rearrangements mediated by insertion elements that have been observed in *S. haemolyticus*. Interestingly, further studies are needed to define the role of *cbp* gene, which was more frequently present in clinically originated strains than nursing house-originated strains.

Several limitations associated with the present study warrant mention. First, only a single species, *S. haemolyticus*, was studied in our work, which may not be representative of the whole CoNS family. Second, some commensals were identified in lung homogenate, suggesting that they were introduced during the inoculation procedure consisting of transglottic tracheal administration. Interaction between CoNS and other microorganisms remains undocumented, and the possibility that they can play a role in lung parenchymal infection cannot be ruled out. Third, several differences exist between mice and humans, precluding the generalization of our findings. Tracheobronchial branching is less complex in mice than in humans. Respiratory bronchioles do not exist in mice, and airways terminate directly into alveolar ducts (Bal and Ghoshal, 1988). Therefore, foci of pneumonia are not centered on infected respiratory bronchioles like in humans and infection disseminates more easily in the lung parenchyma. In humans, bronchial submucosal glands secrete lysozyme, which keeps the lower respiratory tract sterile. Submucosal glands are absent from the conducting airways in mice (Skerrett, 2004), reducing local anti-infectious defenses. Circulating neutrophil counts are lower in mice than in humans and lack α-defensins (Eisenhauer and Lehrer, 1992), whereas natural killer cell activity is generally higher in murine than in human lungs (Matute-Bello et al., 2011). These species differences may facilitate or protect against *S. haemolyticus* virulence. Also, exploring other signaling pathways such as the anti-inflammatory side of the immune response may help better explain the underlying mechanisms of the pathophysiology of this *S. haemolyticus*-induced pneumonia.

## Conclusion

This study provides evidence that tracheal inoculation of *S. haemolyticus* to immunocompetent mice produces interstitial and confluent pneumonia of moderate severity with a rapid and spontaneous recovery of inflammatory markers and clinical condition. Moreover, *icaA* and *icaD* independent biofilm formation is a common phenotype in *S. haemolyticus* isolates.

## Data Availability Statement

All datasets generated for this study are included in the manuscript/Supplementary Files.

## Ethics Statement

The animal study was reviewed and approved by the Ruijin Hospital Ethics Committee, Shanghai Jiao Tong University School of Medicine.

## Author Contributions

M-MS and Y-PX contributed to the conception and design, the collection and/or assembly of data, the data analysis and the interpretation, and the writing of the manuscript. AM and J-JR contributed to the conception and design, the data analysis and the interpretation, and the writing of the manuscript. Y-GZ and J-MQ contributed to the conception and design, financial support, data analysis and interpretation, the writing of the manuscript, and final approval of the manuscript.

## Conflict of Interest

The authors declare that the research was conducted in the absence of any commercial or financial relationships that could be construed as a potential conflict of interest.

## Footnotes

1 http://www.beyotime.com/product/P0010.htm

2 http://www.furi.com.cn
