# Supplementary figures and images for: Inoculation Pneumonia Caused by Coagulase Negative Staphylococcus
Source: Front Microbiol. 2019 Oct 4;10:2198. doi: 10.3389/fmicb.2019.02198 (PMC6787291; doi:10.3389/fmicb.2019.02198)

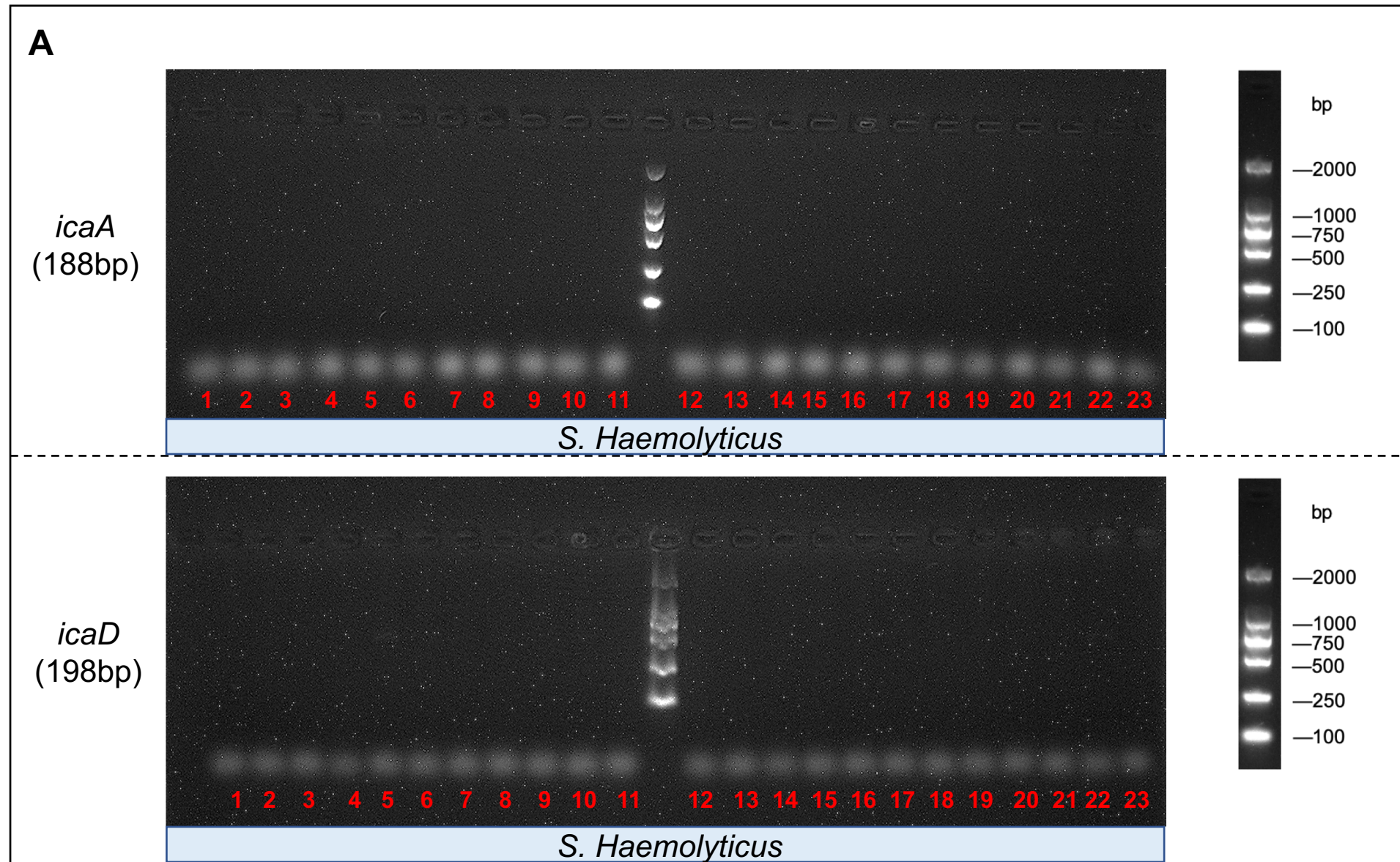

**Fig.S1**

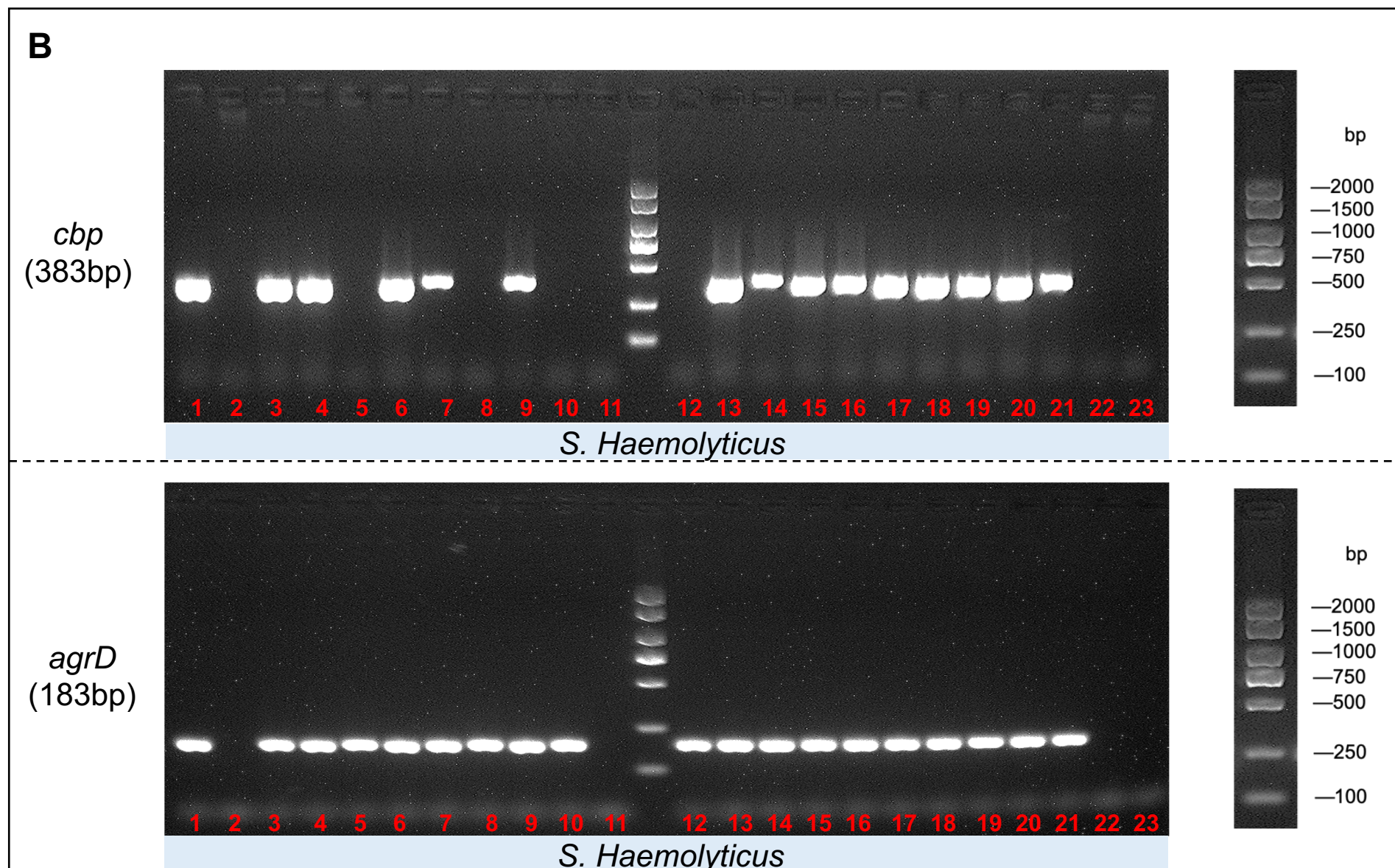

**Fig.S1**

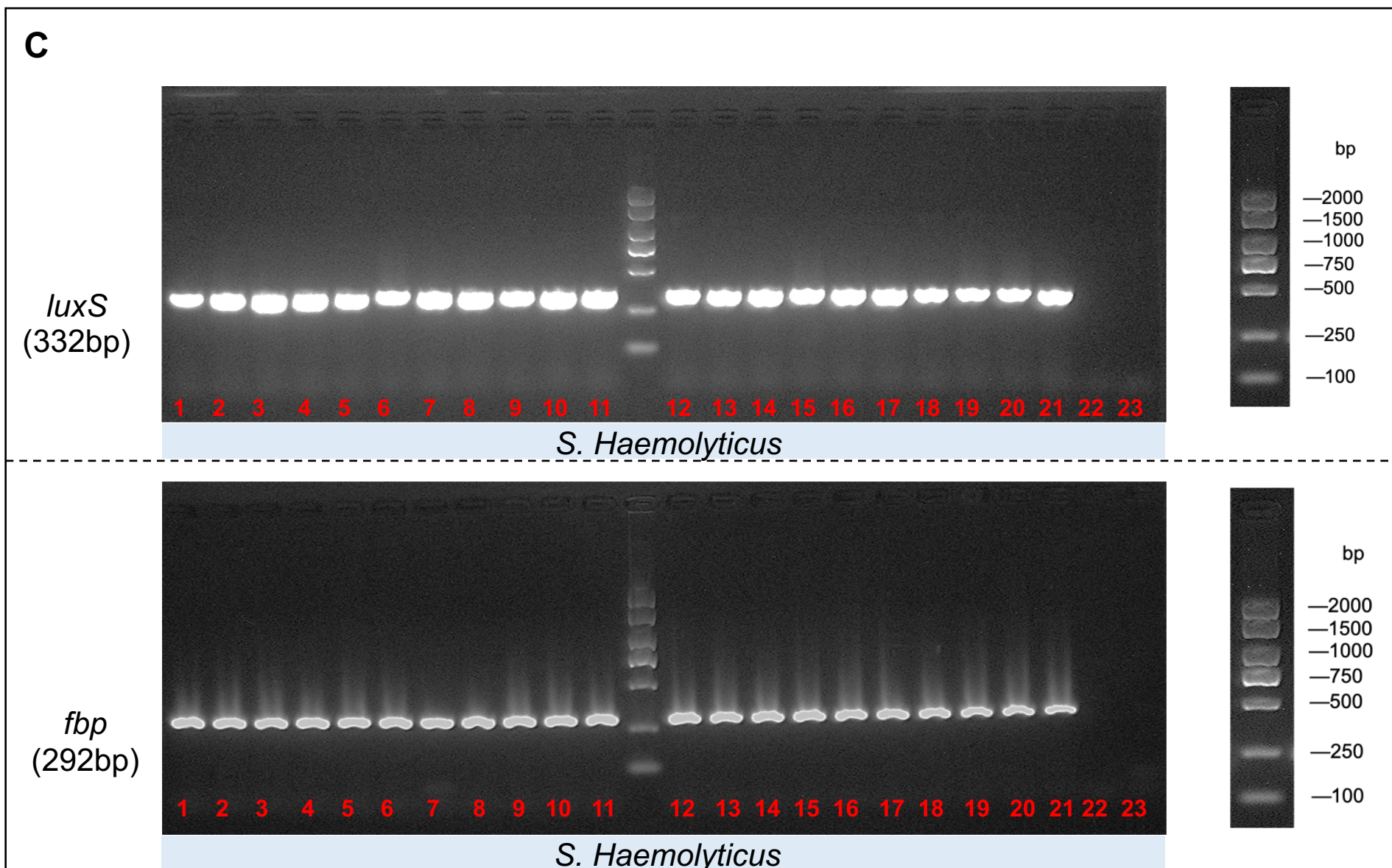

**Fig.S1**

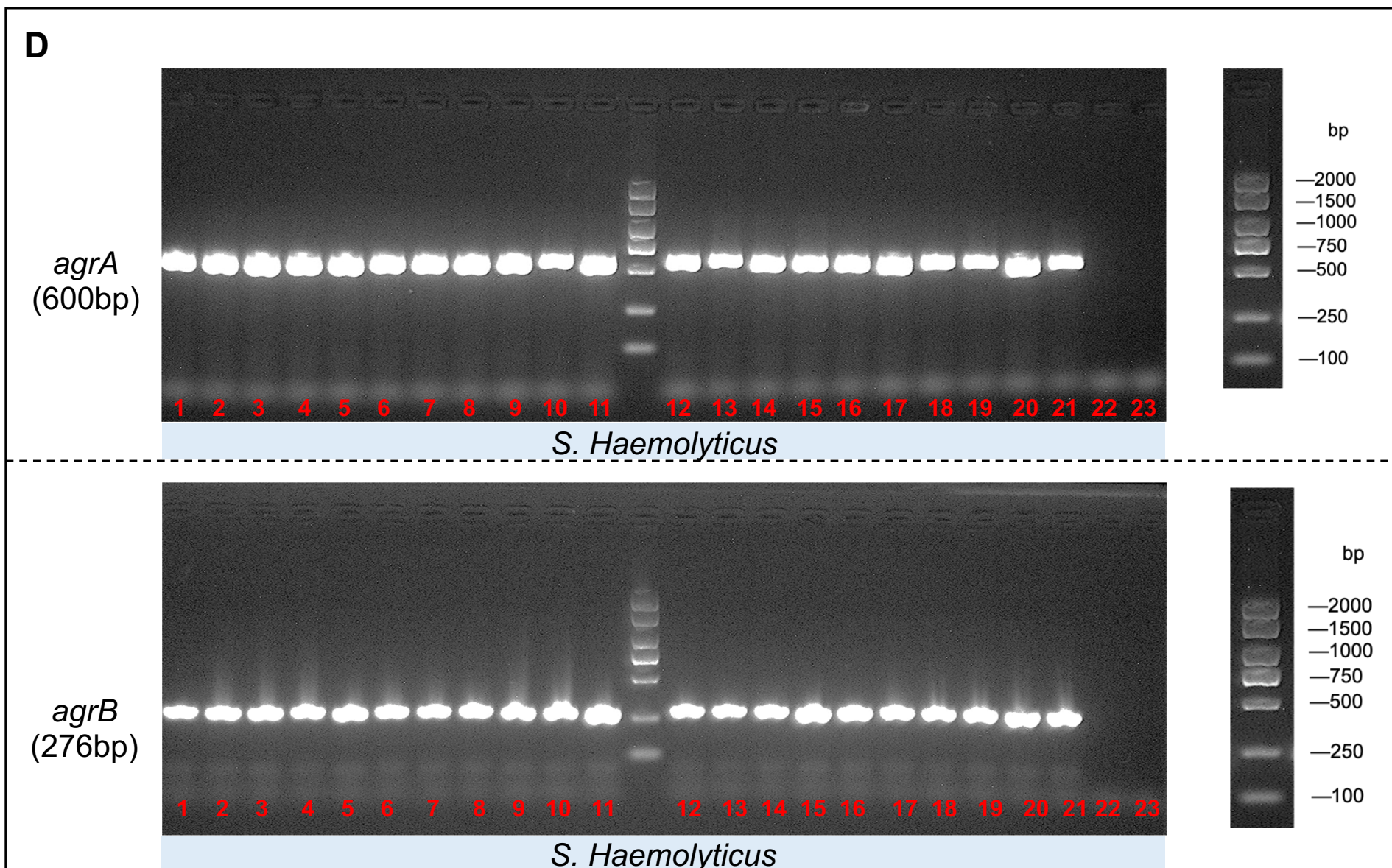

**Fig.S1**

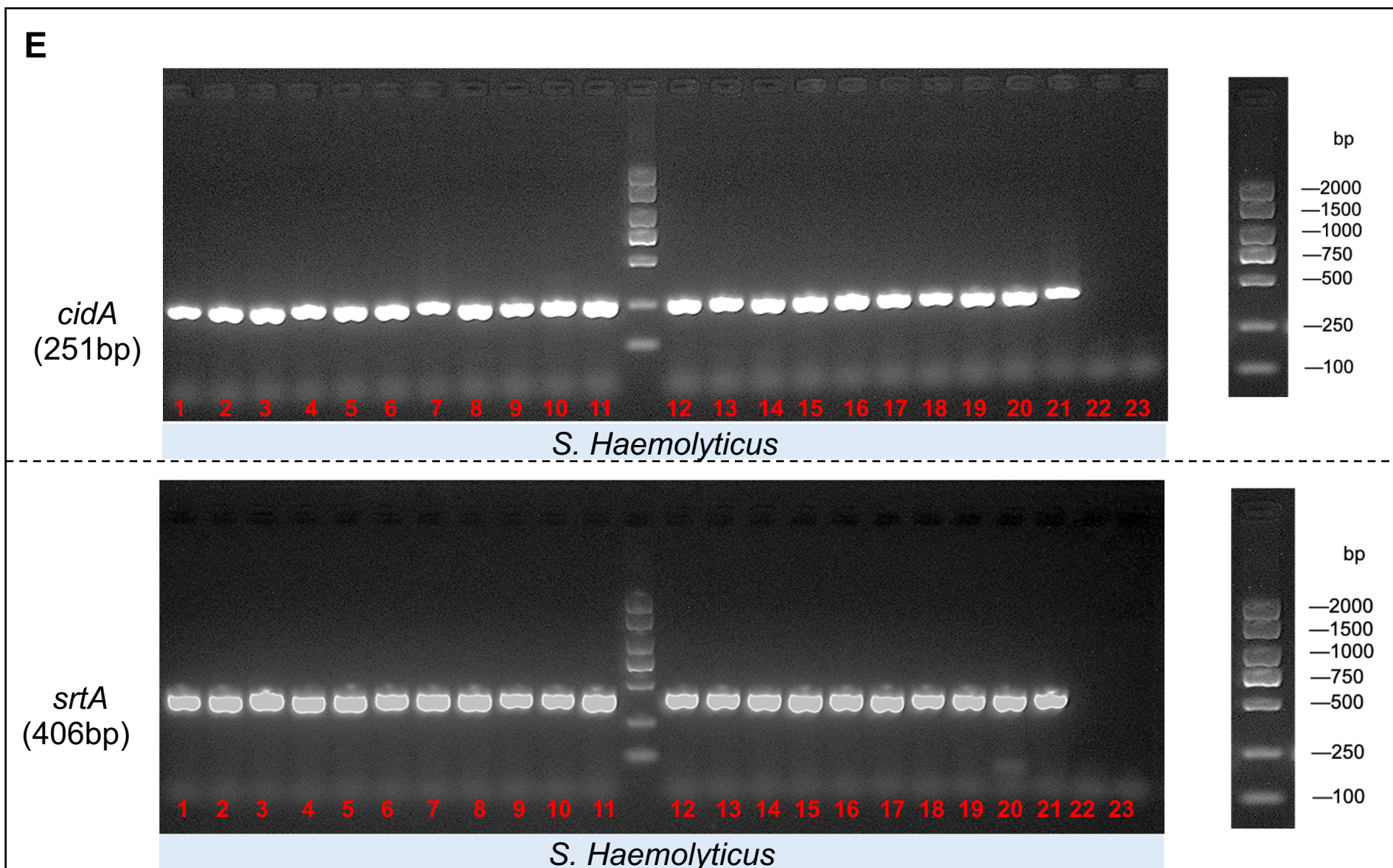

**Fig.S1**

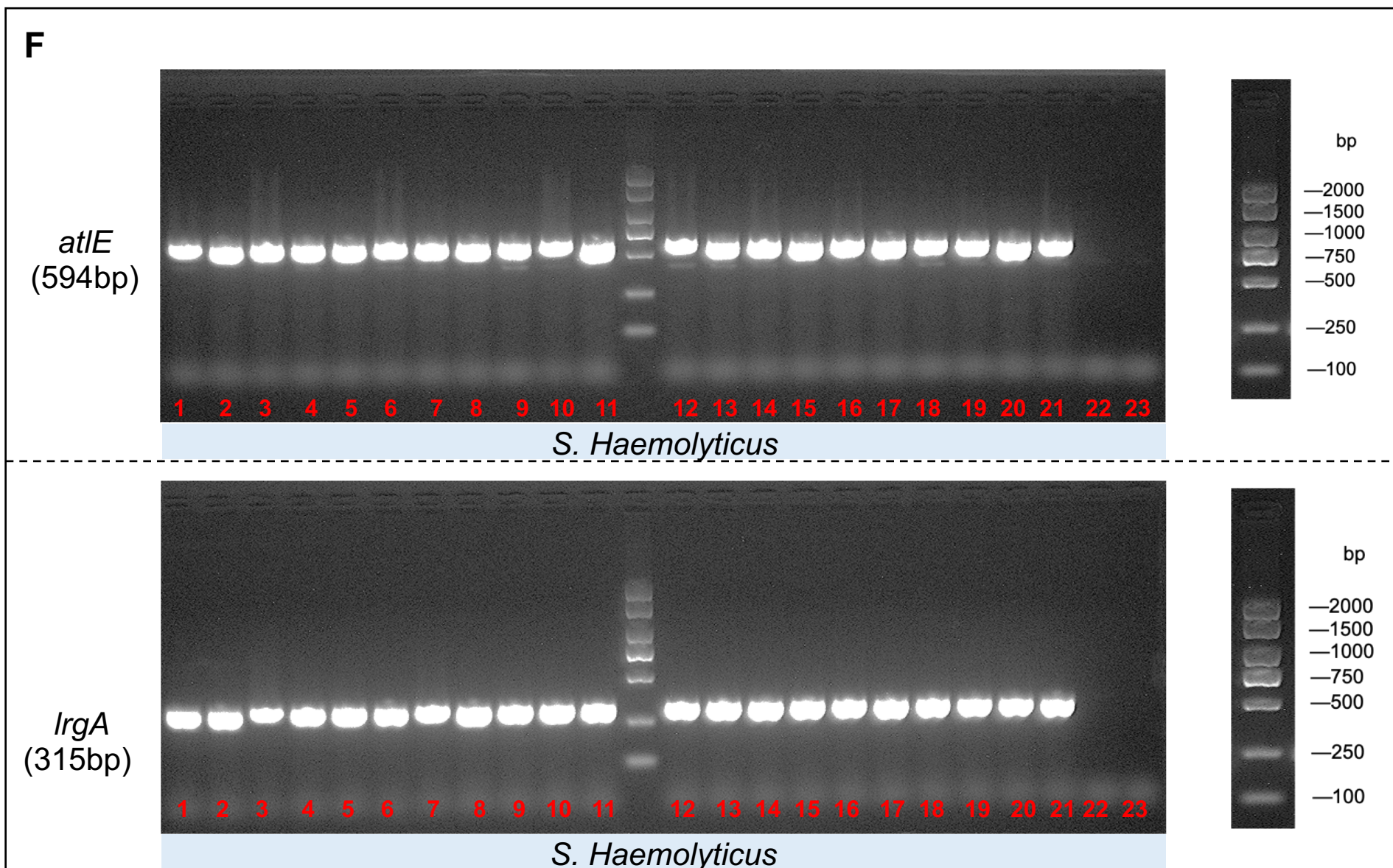

**Fig.S1**

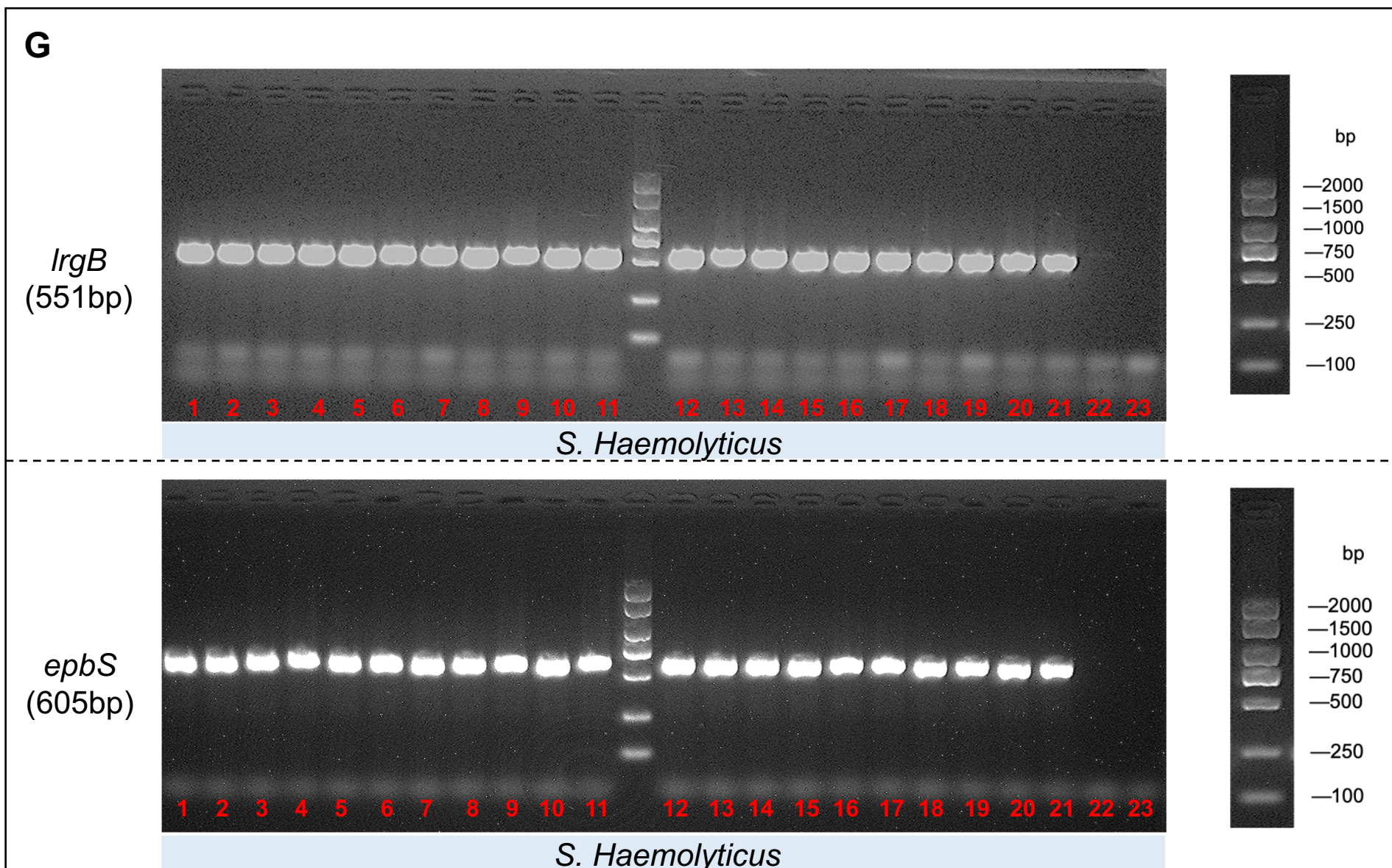

**Fig.S1**

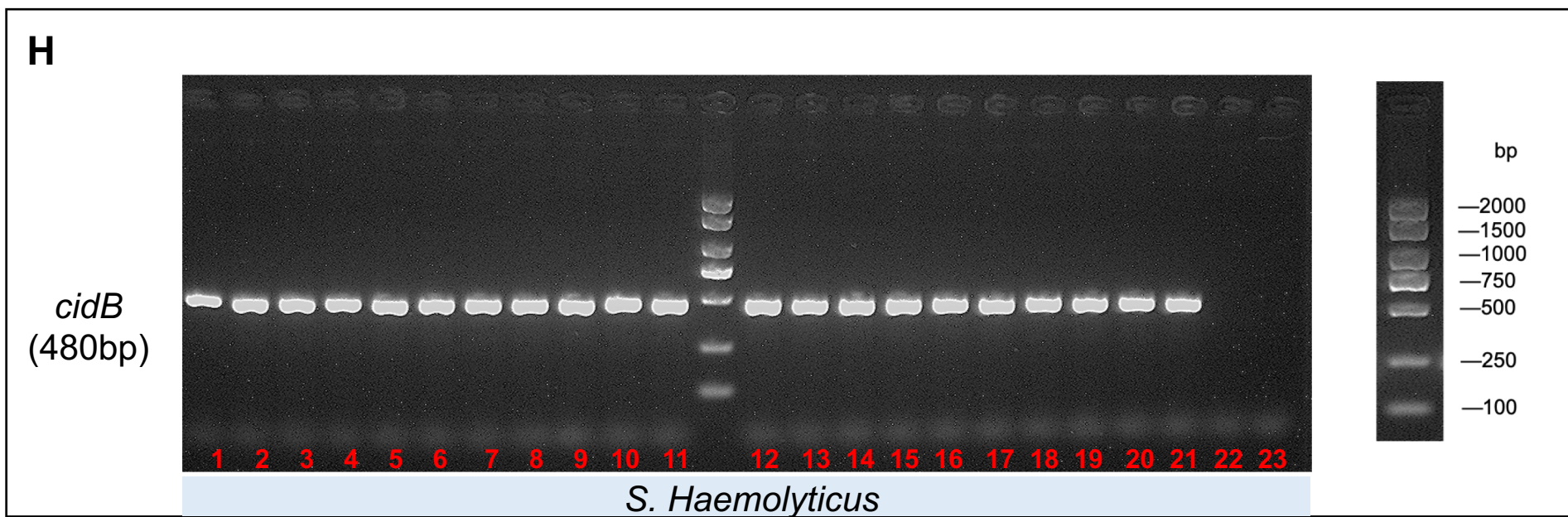

**Fig.S1**

Supplement: Supplementary file 1 [file Data_Sheet_1.PDF]
