# Supplementary material for: Inoculation Pneumonia Caused by Coagulase Negative Staphylococcus
Source: Front Microbiol. 2019 Oct 4;10:2198. doi: 10.3389/fmicb.2019.02198 (PMC6787291; doi:10.3389/fmicb.2019.02198)
Supplement: Supplementary file 2 [file Table_1.pdf]

## On-line Data Supplement

**Supplementary Table S1**

Lung injury scoring system

| Parameter | Score per field | | |
|---|---|---|---|
| | 0 | 1 | 2 |
| A. Neutrophils in the alveolar space | None | 1-5 | >5 |
| B. Neutrophils in the interstitial space | None | 1-5 | >5 |
| C. Hyaline membranes | None | 1 | >1 |
| D. Proteinaceous debris filling the airspaces | None | 1 | >1 |
| E. Alveolar septal thickening | <2x | 2x-4x | >4x |

Score = [(20 × A) + (14 × B) + (7 × C) + (7 × D) + (2 × E)] / (number of fields × 100)

**Supplementary Table S2**

Primer sequences

| **Target** | **Primers** | **Sequence(5'-3')** | **Amplicon size(bp)** |
|---|---|---|---|
| icaA | F | TCTCTTGCAGGAGCAATCAA | 188 |
| | R | TCAGGCACTAACATCCAGCA | |
| icaD | F | ATGGTCAAGCCCAGACAGAG | 198 |
| | R | CGTGTTTTCAACATTTAATGCAA | |
| lrgA | F | CCCTCCCCAAGTTTAGTTTGTT | 315 |
| | R | ATTTCCTATGCCAGCGTCAGT | |
| lrgB | F | GGTGGAACAATGGCAACGAT | 551 |
| | R | ATGGTTGCGGGGATCGTATT | |
| agrA | F | CCGAAGCAAAACAGTGCTCA | 600 |
| | R | AGATTGCGCTTGCAACAGAC | |
| agrB | F | GCTTAAACGGTTCTGGTGCT | 276 |
| | R | ACACGGTGCACATGCAAAAT | |
| agrD | F | ACGTGCGATTAAAGGCTTGG | 183 |
| | R | AACGCATCTTCAAGTGCTTCAG | |

| | | | |
|---|---|---|---|
| luxS | F | GCCCAACCGCATTGTACTTC | 332 |
| | R | GTTGTTGCACCTTTCGTCCG | |
| ebpS | F | TTCCCACCTAGAAACGCACA | 605 |
| | R | TTAGCGTCACGATGGTCGTC | |
| fbp | F | CCACCAATGTTTGCTCGTGT | 292 |
| | R | TGTTTTGAGTAGGTGGCGCT | |
| cbp | F | TACGCTAGCGCAAGGTGAAT | 383 |
| | R | TGTTTAACGGACGGCCAACT | |
| atlE | F | GCAAAGGTGGTCCTGCTACT | 594 |
| | R | GGTAAGCGTGTGGGTCAGAA | |
| cidA | F | CTAGCTGGAAGTATCGTAGGCA | 251 |
| | R | ACCATYTTTTCAGCGATGTAACC | |
| cidB | F | CCCAGCGTTAGTAGGTTCGG | 480 |
| | R | TTTGCGGTACCRAAAGCGTG | |
| srtA | F | AAACAAACACCAACCATCCCT | 406 |
| | R | CCCAAACACCGGTTTGTTCAT | |

**Supplementary Table S3**

Details of *S. haemolyticus*

| Lane No. | Strain | Source | Specimen origin |
|---|---|---|---|
| 1 | *S. haemolyticus* | nursing house | nasal swabs |
| 2 | *S. haemolyticus* | nursing house | nasal swabs |
| 3 | *S. haemolyticus* | nursing house | axillary swabs |
| 4 | *S. haemolyticus* | nursing house | axillary swabs |
| 5 | *S. haemolyticus* | nursing house | axillary swabs |
| 6 | *S. haemolyticus* | nursing house | axillary swabs |
| 7 | *S. haemolyticus* | nursing house | axillary swabs |
| 8 | *S. haemolyticus* | nursing house | axillary swabs |
| 9 | *S. haemolyticus* | nursing house | axillary swabs |
| 10 | *S. haemolyticus* | nursing house | nasal swabs |
| 11 | *S. haemolyticus* | hospital | blood |
| | Marker | | |

| | | | |
|---|---|---|---|
| 12 | *S. haemolyticus* | hospital | blood |
| 13 | *S. haemolyticus* | hospital | blood |
| 14 | *S. haemolyticus* | hospital | blood |
| 15 | *S. haemolyticus* | hospital | blood |
| 16 | *S. haemolyticus* | hospital | blood |
| 17 | *S. haemolyticus* | hospital | blood |
| 18 | *S. haemolyticus* | hospital | blood |
| 19 | *S. haemolyticus* | hospital | blood |
| 20 | *S. haemolyticus* | hospital | blood(for vivo experiment) |
| 21 | *S. haemolyticus* ATCC29970 | | |
| 22 | *S. epidermidis* ATCC12228 | | |
| 23 | Negative | | |

**Supplementary Table S4**

Antimicrobial Resistance Profile of *S. haemolyticus*

| | Lane No. | | | | | | | | | |
|---|---|---|---|---|---|---|---|---|---|---|
| | 11 | 13 | 14 | 15 | 16 | 17 | 18 | 19 | 20 | 21 |
| P | R | R | R | R | R | R | R | R | R | R |
| OX | R | R | R | R | S | R | S | R | R | R |
| CIP | S | R | R | R | S | R | S | S | R | R |
| VA | S | S | S | S | S | S | S | S | S | S |
| TE | S | S | S | R | R | S | S | S | S | S |
| DA | R | R | R | R | S | R | R | S | R | S |
| SXT | S | S | R | S | S | R | S | S | S | R |
| MXF | S | I | I | R | S | R | S | S | R | I |
| RD | S | S | S | S | S | S | S | S | S | S |
| E | R | R | R | R | S | R | S | S | R | S |
| CN | S | I | R | R | S | R | S | S | R | S |
| LEV | S | R | R | R | S | R | S | S | R | R |
| LZD | S | S | S | S | S | S | S | S | R | S |

P, Penicillin; OX, Oxacillin; CIP, Ciprofloxacin; VA, Vancomycin; TE, Tetracycline; DA, Clindamycin; SXT, Sulfamethoxazole/Trimethoprim; MXF, Moxifloxacin; RD, Rifampicin; E,

Erythromycin; CN, Gentamicin; LEV, Levofloxacin; LZD, Linezolid.

**Supplementary FigureS1**

(A-H) Gel electrophoresis of multiplex PCR showed fragments of *icaA, icaD, cbp, agrD*, *ebpS, fbp, srtA, agrA, agrB, luxS, cidA, cidB, lrgA, lrgB* and *atlE* for *S. haemolyticus*. Lanes 1 to 10 are strains from nursing houses. Lanes 11 to 19 are strains from hospitals. Lane 20 is the *S. haemolyticus* used in our vivo experiment. Lane 21 is *S. haemolyticus* ATCC29970. Lane 22 is *S. epidermidis* ATCC12228. Lane 23 is reaction negative control.

**Supplementary Video**

Mice home cage activities were observed at 12 and 36 hours after inoculation of $10^6$, $10^7$ or $10^8$ CFU of *S. haemolyticus*, respectively. The video loop of inoculation of $10^8$ at 12 hours showed markedly altered behavioral performance, such as horizontal locomotion, climbing the cage lid, hanging on the lid and jumping, when compared with other groups.
